# Endotheliopathy in CAR T-Cell Therapy: Mechanistic Insights into the VWF/ADAMTS13 Axis and the Angiopoietin–Tie2 Pathway

**DOI:** 10.1055/a-2923-1208

**Published:** 2026-08-11

**Authors:** Sévérine de Bruijn, Inge Vangenechten, Sébastien Anguille, Alain Gadisseur

**Affiliations:** 1 Department of HaematologyAntwerp University HospitalEdegemAntwerpBelgium; 2 Haemostasis and Thrombosis Research CenterAntwerp University HospitalEdegemAntwerpenBelgium

**Keywords:** CAR T-cell therapy, immunothrombosis, coagulopathy, von Willebrand factor, ADAMTS13, angiopoietins, CRS, ICANS

## Abstract

Chimeric antigen receptor (CAR) T cell therapy has transformed the management of hematologic malignancies, yet its clinical success is tempered by severe immune-mediated toxicities, including cytokine-release syndrome (CRS) and immune effector cell–associated neurotoxicity syndrome (ICANS), often accompanied by CAR T cell therapy-related coagulopathy (CARAC). Converging evidence identifies therapy-related endotheliopathy as a central pathophysiological link between cytokine excess, hemostatic dysregulation, capillary leak, and organ injury. Parallel efforts aim to identify circulating biomarkers that can signal emerging toxicity before clinical deterioration. This review summarizes the biological basis of endotheliopathy during CAR T cell therapy, with particular emphasis on two interconnected regulatory systems: the von Willebrand factor (VWF)/ADAMTS13 axis, which governs platelet adhesion and microvascular thrombosis, and the angiopoietin (Ang)–tyrosine kinase receptor Tie2 signaling pathway, which regulates endothelial stability and vascular permeability. Dysregulation of these pathways drives the shift from adaptive immunothrombosis to pathological endothelial injury, characterized by loss of anticoagulant control, barrier disruption, and microvascular instability. Clinical studies show that alterations in the VWF/ADAMTS13 balance and increases in the Ang-2/Ang-1 ratio correlate with CRS and ICANS severity and may precede overt toxicity, highlighting their potential as markers of endothelial vulnerability. Defining actionable biomarker thresholds and evaluating endothelial-targeted interventions are key priorities for improving the safety and precision of CAR T cell therapy.

## Introduction

Chimeric antigen receptor (CAR) T cell therapy has revolutionized the treatment of advanced hematological cancers, offering hope to patients with relapsed or refractory diseases. 1 2 By genetically engineering autologous T lymphocytes to target specific tumor-associated antigens—such as CD19 or B cell maturation antigen (BCMA)—this adoptive immunotherapy redirects cytotoxicity toward malignant cells to induce deep, durable clinical responses. 3 4 Despite its remarkable efficacy, CAR T cell therapy is associated with potentially life-threatening immune-mediated toxicities. The most prominent of these are cytokine-release syndrome (CRS), immune effector cell–associated neurotoxicity syndrome (ICANS), and secondary CAR T cell therapy-related coagulopathy (CARAC), which together represent major contributors to treatment-related morbidity and, in severe cases, mortality. 5 6 7 8 9 10 While tumor-debulking bridging therapies and early supportive care have mitigated some risks, nonrelapse mortality remains significant and varies considerably across different CAR constructs. 11 12

Elucidating how immune activation drives organ injury is a clinical priority, with growing evidence identifying the vascular endothelium as the critical interface between systemic inflammation and microvascular dysfunction during CAR T cell therapy. 13 14 15 16 17 18 19 Far from being a passive barrier, endothelial cells actively coordinate inflammatory and hemostatic responses. Systemic cytokine signaling triggers endothelial activation, accelerating Weibel–Palade body (WPB) exocytosis, the upregulation of adhesion molecules, complement engagement, and neutrophil extracellular trap (NET) formation. Combined, these responses give rise to immunothrombosis, an intravascular defense mechanism that spatially confines inflammatory stimuli within the microvasculature. When inflammatory signaling is sustained or amplified, however, this protective response may evolve into pathological endotheliopathy, marked by barrier disruption, increased vascular permeability, and a prothrombotic endothelial phenotype. 20 21 22 23 24

While these processes are well established in inflammatory conditions such as sepsis, COVID-19, and posttransplant vascular syndromes, their precise contribution to CAR T cell–associated toxicities is only beginning to be uncovered. Within this context, endothelial-derived biomarkers have emerged as promising tools for monitoring and potentially predicting treatment-related complications. Research highlights two interconnected regulatory systems central to this vascular toxicity. The first is the von Willebrand factor (VWF)/ADAMTS13 axis, which regulates platelet adhesion and microvascular thrombosis. The second is the angiopoietin (Ang)–tyrosine kinase receptor Tie2 signaling pathway, which governs endothelial stability and vascular permeability. Dysregulation of these pathways may represent the molecular bridge linking cytokine excess to the vascular manifestations of CRS, ICANS, and CARAC. 16 17 18 19 25 26 27 28 Although these candidate biomarkers remain investigational and lack standardized thresholds, they offer important mechanistic insight and may ultimately support risk-adapted interventions.

This review bridges the gap between separate vascular and immuno-hemostatic models by examining how the VWF/ADAMTS13 axis and the Ang–Tie2 pathway collectively drive CRS, ICANS, and CARAC. By consolidating the biological basis of this shared endotheliopathy, this text outlines biomarker-guided risk stratification and introduces emerging endothelial-targeted strategies to improve clinical outcomes.

## Endothelial Activation and Immunothrombosis as Drivers of CAR T Cell Toxicity

### Physiological Endothelial Regulation and Immunothrombosis

The innate immune system and hemostasis are tightly interconnected biological networks. Beyond preventing blood loss, the coagulation cascade functions as an effector arm of innate immunity during infection and tissue injury. 20 21 22 23 24 Under physiological conditions, the vascular endothelium maintains this delicate balance through the continuous release of anticoagulant and anti-inflammatory mediators, including nitric oxide (NO), prostacyclin (PGI _2_ ), thrombomodulin (TM), tissue-type plasminogen activator, and tissue factor pathway inhibitor (TFPI). 22 29 NO downregulates endothelial adhesion molecules involved in leukocyte rolling and firm adhesion, including P-selectin, E-selectin, vascular cell adhesion molecule 1 (VCAM-1), and intercellular adhesion molecule-1 (ICAM-1), thereby limiting leukocyte recruitment and platelet adhesion, while PGI _2_ restrains platelet activation and leukocyte extravasation. Collectively, these mechanisms preserve endothelial quiescence and protect the vasculature from inappropriate thrombus formation. 22 30 31 32

During inflammatory stress, this equilibrium shifts toward a pro-inflammatory and prothrombotic phenotype. Cytokines including interleukin (IL)-1, IL-6, and tumor necrosis factor-α (TNF-α) activate endothelial cells through mitogen-activated protein kinase/nuclear factor kappa-light-chain enhancer of activated B cell (MAPK/NFκB) signaling pathway, a state often referred to as type II endothelial activation. 22 33 One of the earliest responses is the exocytosis of WPBs, which releases ultra-large von Willebrand factor (ULVWF) multimers and P-selectin into the circulation. 29 30 34 At the same time, endothelial cells upregulate tissue factor (TF), plasminogen activator inhibitor-1 (PAI-1), and adhesion molecules such as ICAM-1 and VCAM-1, promoting leukocyte recruitment and local coagulation activation. Disruption of endothelial junctions, reflected by increased platelet endothelial cell adhesion molecule-1 (PECAM-1), further facilitates leukocyte and fluid extravasation. 22 30 33 34 35

Activated neutrophils amplify endothelial activation through formation of NETs, web-like chromatin structures decorated with histones and proteolytic enzymes. NETs provide a scaffold that binds ULVWF multimers and recruits platelets, while also activating factor XII and inhibiting endogenous anticoagulants such as TFPI. In addition, histones released within NETs can directly injure endothelial cells, which further drives thrombo-inflammatory signaling within the microvasculature. 21 23 33 36

**Fig. 1:**
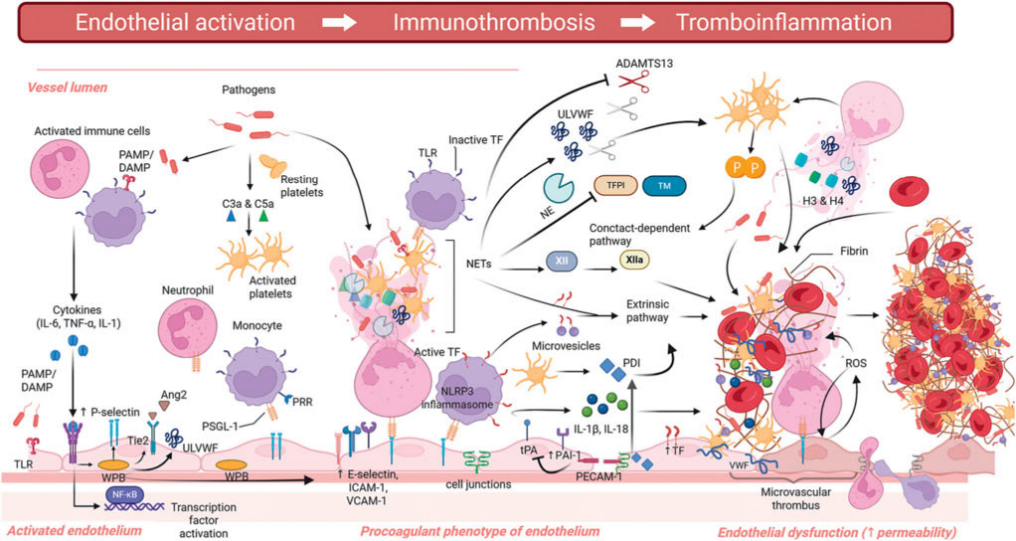
Cellular and molecular mechanisms of immunothrombosis. Immunothrombosis represents a physiological intravascular defense mechanism in which endothelial activation, platelets, monocytes, and neutrophils cooperatively generate localized fibrin- and VWF-rich microthrombi to contain pathogens. Activated endothelial cells release VWF and upregulate TF, while platelets provide procoagulant surfaces and release polyphosphates that enhance coagulation. Neutrophils form NETs, chromatin lattices enriched with histones and neutrophil elastase, which bind VWF, activate FXII, and inhibit endogenous anticoagulants such as TFPI, thereby amplifying platelet recruitment and thrombin generation. Together, these processes confine infection while linking innate immunity to coagulation. (Adapted with permission from De Bruijn (2026) https://BioRender.com/kbe9qck ; created in BioRender.) ADAMTS13, a disintegrin-like and metalloproteinase with thrombospondin type-1 motifs, member 13; DAMPs, damage-associated molecular patterns; ICAM-1, intercellular adhesion molecule 1; NE, neutrophil elastase; NF-kB, nuclear factor kappa B; P, polyphosphate; PAMPs, pathogen-associated molecular patterns; PDI, protein disulphide isomerase; PECAM-1, platelet endothelial cell adhesion molecule-1; PSGL-1, P-selectin glycoprotein ligand-1; ROS, reactive oxidative species; TF, tissue factor; TFPI, tissue factor pathway inhibitor; TM, thrombomodulin; TLR, Toll-like receptor; ULVWF, ultra-large von Willebrand factor multimers; VCAM-1, vascular cell adhesion molecule 1; VWF, von Willebrand factor.

The coordinated interaction between endothelial activation, ULVWF multimers release, platelet recruitment, and NET formation results in immunothrombosis, a physiological mechanism that confines inflammatory stimuli within the microvasculature. 23 36 When appropriately regulated, immunothrombosis is protective. However, if inflammatory stimuli persist or intensify, these same pathways lose their spatial restriction and evolve into systemic thrombo-inflammation, a maladaptive state marked by reduced TM, TFPI, and ADAMTS13 activity, heightened TF expression, and progressive endotheliopathy. 22 24 37 38 39 40 41 These vascular alterations therefore represent a key step in the transition from defensive immunothrombosis to pathological thrombo-inflammation ( Fig. 1 ).

### VWF/ADAMTS13 and Angiopoietins in Endothelial Dysregulation

VWF is a large multimeric glycoprotein positioned at the interface of hemostasis, inflammation, and vascular integrity. Synthesized by endothelial cells and megakaryocytes and stored in WPBs and platelet α-granules, VWF mediates platelet adhesion to exposed subendothelium under high shear stress and stabilizes circulating factor VIII (FVIII). 42 43 The evolutionary importance of VWF is highlighted by the fact that its quantitative or qualitative defects cause von Willebrand disease, the most common inherited bleeding disorder. 44 Beyond coagulation, however, accumulating evidence demonstrates that VWF regulates leukocyte recruitment, vascular permeability, complement activation, and angiogenesis, identifying it as a multifunctional guardian of vascular homeostasis. 45

The prothrombotic potential of VWF scales with its multimeric length and is strictly regulated by ADAMTS13; this metalloproteinase cleaves highly adhesive ULVWF multimers into smaller, less reactive forms, effectively limiting inappropriate platelet tethering. 46 47 In addition to its proteolytic role, ADAMTS13 exerts anti-inflammatory effects by limiting leukocyte–platelet interactions and dampening cytokine amplification, serving as a vital counter-regulator of endothelial stress. 48 49 50

Inflammatory cytokines profoundly disturb this regulatory balance. IL-6, IL-8, and TNF-α promote WPB exocytosis while simultaneously downregulating ADAMTS13 synthesis and activity, which favors ULVWF persistence. 34 49 50 The resulting imbalance is classically exemplified by thrombotic thrombocytopenic purpura (TTP), 51 and is increasingly recognized across hyperinflammatory states such as sepsis 52 53 and COVID-19. 40 41 54 In these conditions, alterations in the VWF/ADAMTS13 ratio correlate with disease severity and organ dysfunction, establishing this axis as both a mechanistic driver and a biomarker of endothelial stress. Similarly, this thrombo-inflammatory cascade is highly active in allogeneic hematopoietic stem cell transplantation (allo-HSCT) settings, where cytoreductive conditioning uniformly triggers acute endothelial activation, and baseline elevations in functional VWF parameters serve as independent indicators of preexisting endothelial vulnerability to subsequent vascular complications. 55

NETs further potentiate endothelial injury by providing a scaffold for ULVWF multimers and reducing ADAMTS13 activity, thereby stabilizing platelet–VWF aggregates and protecting them from proteolytic cleavage. These effects reinforce platelet recruitment and sustain microvascular thrombosis, creating a self-amplifying NET–VWF–ADAMTS13 circuit that propagates thrombo-inflammatory signaling. 56 57 This integrated pathway has been implicated in complement- and inflammation-driven thrombotic microangiopathies. 58 59

A second key regulatory pathway controlling endothelial stability is the Ang–Tie2 axis. Angs are endothelial growth factor family members that bind the Tie2 receptor, a tyrosine kinase expressed on endothelial cells, and regulate vascular quiescence and barrier integrity. 60 61 Among them, Ang-1 and Ang-2 are the best characterized and exert largely opposing effects on endothelial homeostasis. Under physiological conditions, Ang-1 is constitutively produced by pericytes and vascular smooth muscle cells. Because circulating Ang-1 levels normally exceed those of Ang-2, Tie2 signaling is predominantly driven by Ang-1. Activation of Tie2 stimulates the phosphoinositide-3-kinase–protein kinase B (PI3K–AKT) pathway, which promotes endothelial survival, suppresses adhesion molecule expression such as VCAM-1 and E-selectin, reduces vascular permeability, and strengthens intercellular junctions. 61 62 63 The indispensable nature of Ang-1–Tie2 signaling is illustrated by knockout models, in which loss of either component results in impaired vasculogenesis and embryonic lethality. 63 64 65

**Fig. 2:**
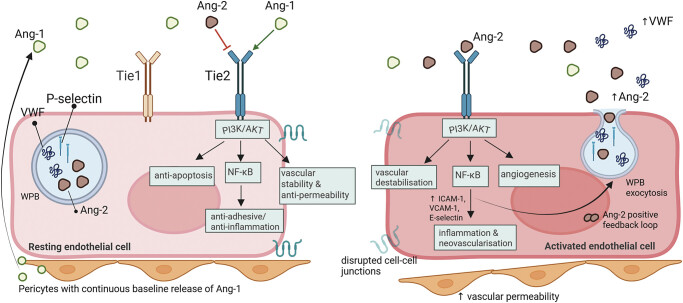
Angiopoietin–Tie2 signaling in endothelial homeostasis and dysfunction. Ang-1-mediated Tie2 activation maintains endothelial quiescence, whereas Ang-2 predominance promotes inflammation, vascular permeability, and endothelial activation. (Adapted with permission from De Bruijn (2026); https://BioRender.com/jc3j4bq ; created in BioRender.) Ang, angiopoietin; NF-κB, Nuclear factor-κB; ICAM-1, intercellular adhesion molecule-1; PI3K-AKT, phosphoinositide-3-kinase-protein kinase (PI3K-AKT) pathway; VCAM-1, vascular cell adhesion molecule 1; VWF, von Willebrand factor.

In contrast, Ang-2 acts as a context-dependent antagonist or partial agonist of Tie2. Synthesized by endothelial cells and stored in WPBs, Ang-2 is rapidly released during inflammatory stimulation. Excess circulating Ang-2 competitively displaces Ang-1 from the Tie2 receptor, inducing rapid endothelial destabilization and a procoagulant, pro-inflammatory state. 60 61 62 Accordingly, an elevated Ang-2/Ang-1 ratio serves as a reliable clinical hallmark of endotheliopathy in inflammatory conditions such as sepsis and acute respiratory distress syndrome. 61 66 67 Although VWF and Ang-2 are co-stored in WPBs, their release is regulated by distinct signaling pathways, and circulating levels do not necessarily correlate. 60 68 A schematic representation of the Ang–Tie2 ligand–receptor pathway and its opposing regulatory effects on endothelial stability is provided in Fig. 2 .

Despite being described as separate systems, the VWF/ADAMTS13 axis and the Ang-Tie2 pathway are biologically interconnected. VWF is essential for the formation of WPBs, the endothelial storage organelles that also contain Ang-2, linking VWF expression directly to endothelial secretory capacity. 69 70 Experimental data further suggest that VWF deficiency can enhance Ang-2 expression through a Tie2–AKT–FOXO1–dependent feedback mechanism, directly coupling primary hemostatic regulation to angiogenic and permeability signaling. 71

Together, these two interlocking pathways orchestrate the shift from vascular quiescence to inflammatory thrombosis. Early disturbances in either system during CAR T cell therapy likely reflect cytokine-induced endothelial stress and may serve as biomarkers of emerging toxicity.

### Endothelial Involvement in CRS and ICANS

#### Cytokine-Driven Endothelial Activation During CAR T Cell Therapy

CRS arises from robust CAR T cell activation upon tumor-associated antigen recognition. Engaged CAR T-cells secrete perforin, granzymes, and high concentrations of pro-inflammatory cytokines, including interferon-γ (IFN-γ) and TNF-α, thereby inducing pyroptotic tumor cell death. 12 72 This inflammatory cell death releases damage-associated molecular patterns (DAMPs) that stimulate macrophages and dendritic cells via pattern-recognition receptors. This myeloid activation amplifies the cytokine cascade, most prominently IL-6 and IL-1β. In parallel, macrophage inflammasome activation promotes caspase-1 activation and the maturation of IL-1β, sustaining systemic inflammation. 73 74 75 76 77

As cytokine concentrations rise, endothelial activation rapidly intensifies. MAPK and NF-κB signaling accelerates WPB secretion, resulting in brisk release of VWF, Ang-2, and P-selectin. Inflammatory signaling simultaneously upregulates endothelial adhesion molecules and destabilizes intercellular junctions. These changes shift the endothelium from a regulated inflammatory responder to a driver of vascular permeability, leukocyte recruitment, capillary leak, and immunothrombosis. This escalating stress response forms the mechanistic link between systemic cytokine excess and the clinical phenotype of CRS, including secondary coagulopathy. 34 78 79 Among the cytokines implicated, IL-6 plays a particularly prominent role, explaining the clinical efficacy of IL-6 receptor blockade with tocilizumab as a cornerstone of CRS management. 6 78 80

**Fig. 3:**
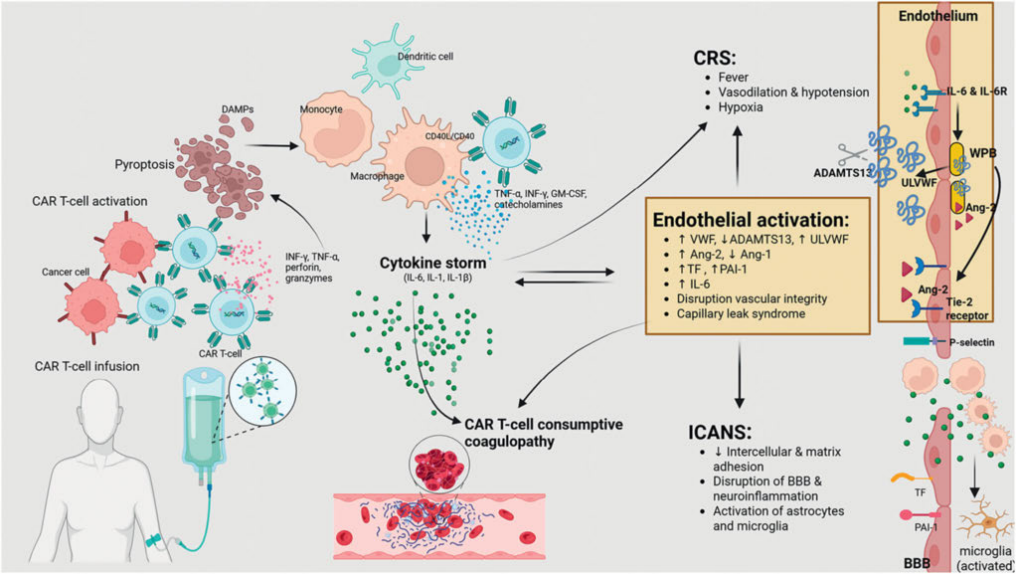
Cytokine-driven endothelial activation and downstream vascular consequences. Immune-mediated cellular stress disrupts vascular tight junctions, triggering the immediate secretion of stored VWF and Ang-2. This loss of endothelial barrier integrity drives a profound shift toward thrombo-inflammation and fluid leakage. Ultimately, these integrated vascular injuries manifest clinically as the systemic capillary leak in CRS and BBB disruption in ICANS. (Adapted with permission from De Bruijn (2026); https://BioRender.com/xcvk52r ; created in BioRender.) ADAMTS13, a disintegrin-like and metalloproteinase with thrombospondin type-1 motifs, member 13; Ang-1, angiopoietin-1; Ang-2, angiopoietin-2; BBB, blood–brain barrier; CAR, chimeric antigen receptor; DAMPs, damage-associated molecular patterns; GM-CSF, granulocyte–macrophage colony-stimulating factor; ICANS, immune effector cell–associated neurotoxicity syndrome; IFN-γ, interferon-gamma; IL-1, interleukin-1; IL-6, interleukin-6; IL-6R, interleukin-6 receptor; NO, nitric oxide; PAI-1, plasminogen activator inhibitor-1; TF, tissue factor; Tie-2, tyrosine kinase with immunoglobulin-like and EGF-like domains 2; TNF-α, tumor necrosis factor-α; ULVWF, ultra-large von Willebrand factor multimers.

**Fig. 4:**
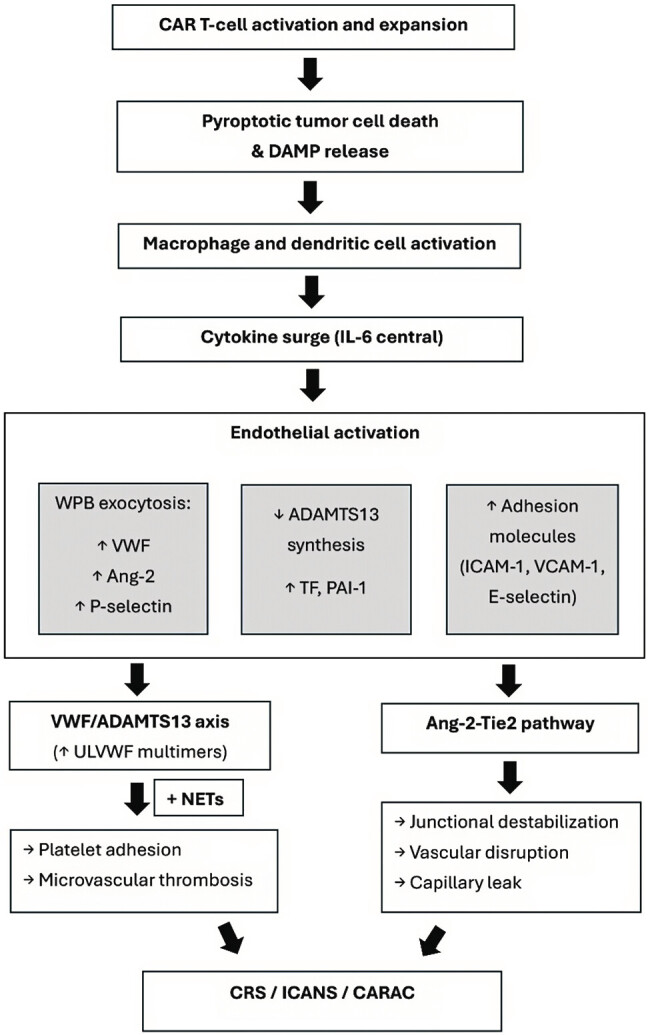
Mechanistic model of endothelial regulatory pathway imbalance in CAR T cell therapy. Cytokine-mediated endothelial activation promotes VWF release, ADAMTS13 insufficiency, and Ang-2 predominance, resulting in thrombo-inflammation, vascular leak, and ultimately CRS, ICANS, and consumptive coagulopathy.

Clinically, CRS is the most frequent early toxicity following CAR T cell infusion, typically emerging within the first 2 weeks. Its presentation ranges from isolated fever to severe systemic inflammation characterized by hypotension, vascular leak, and multiorgan dysfunction. Although CRS is generally reversible, severe cases frequently require intensive care support. 81 82 This timing and potential severity underscore the need to identify early indicators of endothelial stress prior to overt clinical deterioration. 1 6 72 The sequence of cytokine-driven endothelial activation and its downstream vascular consequences is schematically illustrated in Figs. 3 and 4 .

Even though ICANS is regarded as a distinct clinical entity, it frequently co-occurs with CRS, sharing a common pathophysiological basis in therapy-related endotheliopathy that extends beyond the systemic circulation to specialized vascular beds. Within the neurovascular unit, damaged endothelial cells and pericytes compromise blood–brain barrier (BBB) integrity, permitting circulating cytokines and immune cells to infiltrate the central nervous system (CNS). 72 73 83 Myeloid-derived cytokines (such as IL-1 and IL-6) drive standard endothelial activation and decrease tight junction expression, but recent single-cell sequencing has additionally unveiled a critical “on-target, off-tumor” mechanism targeting pericytes. 84 85 Human brain pericytes, which position themselves at endothelial tight junctions to maintain neurovascular integrity, express CD19 and its obligatory chaperone CD81 throughout adulthood. Consequently, CD19-directed CAR T-cells directly target and deplete these cells, provoking the local release of IL-6 and vascular endothelial growth factor (VEGF). These mediators activate adjacent endothelial cells, amplifying neurovascular injury via a positive feedback loop. 84 86 Although the relative contribution of direct pericyte targeting versus systemic cytokine-mediated injury remains debated, this off-target vulnerability helps explain why ICANS occurs with a higher frequency and severity in CD19-targeted therapies compared with constructs targeting alternative antigens (e.g., CD22 or BCMA).

In addition to direct pericyte injury, inflammatory mediators further compromise BBB integrity. Cytokines released during CAR T cell activation, most prominently IFN-γ from CAR T-cells and colony-stimulating factors from monocytes and macrophages, induce endothelial adhesion molecules such as ICAM-1 and VCAM-1, thereby facilitating leukocyte adhesion and transmigration across the compromised barrier. 81 86 Within the CNS, infiltrating cytokines activate microglia and astrocytes, amplifying local inflammatory circuits and contributing to the characteristic neurological manifestations of ICANS. 77 87 Clinical correlates of this neurovascular breakdown include elevated cerebrospinal fluid (CSF) protein, increased CSF leukocyte counts, and in some cases detectable CAR T-cells, although the precise contribution of intracerebral CAR T cell infiltration to neurotoxicity remains uncertain. 6 18 25 73

This systemic-to-central trafficking is further influenced by therapeutic interventions. Interestingly, while IL-6 receptor blockade with tocilizumab effectively controls systemic CRS symptoms, it prevents receptor binding and clearance, leading to a temporary surge in circulating peripheral IL-6 levels. 18 88 89 Whether this sudden spike crosses a compromised BBB and contributes to subsequent neuroinflammation or delayed ICANS remains a subject of ongoing clinical debate. This limitation underscores preclinical observations highlighting the potential primacy of myeloid-derived IL-1 in ICANS pathogenesis. Indeed, upstream receptor blockade with anakinra, unlike tocilizumab, has demonstrated protection against delayed lethal neurotoxicity and BBB disruption in preclinical models. 89 90

ICANS typically emerges within 4 to 10 days after CAR T cell infusion, usually in temporal association with CRS. Higher CRS grades strongly correlate with the risk of severe neurotoxicity following CD19-directed CAR T cell therapy. 5 6 Clinically, ICANS spans a broad spectrum from mild confusion and expressive aphasia to seizures and, in rare instances, cerebral edema. 9 18 25 74 76 77

#### Endothelial Biomarkers and Clinical Manifestations

##### Composite Indices

Given the pivotal role of endothelial injury in the pathogenesis of CRS and ICANS, considerable effort has been directed toward identifying biomarkers that reflect early endothelial stress. Conventional laboratory parameters such as elevated C-reactive protein (CRP), ferritin, D-dimer levels, and thrombocytopenia reflect systemic inflammation and microvascular activation but lack specificity for endotheliopathy. 88 91 To address this limitation, composite indices have been developed.

The Endothelial Activation and Stress Index (EASIX), calculated as (LDH × creatinine)/platelet count, was originally validated in allo-HSCT to predict a continuum of hyperinflammatory endothelial complications, including sinusoidal obstruction syndrome, transplant-associated thrombotic microangiopathy (TMA), engraftment syndrome, and acute graft-versus-host disease. 92 93 94 95 96 97 Recent European Society for Blood and Marrow Transplantation consensus guidelines emphasize that this spectrum of vascular toxicities and coagulation disorders extends directly to advanced cellular therapies. 98 Elevated preinfusion EASIX scores have since been associated with the development and severity of CAR T cell–related toxicities. 27 28 99 Several adaptations aim to improve clinical applicability. The modified EASIX (mEASIX), which replaces creatinine with CRP, has demonstrated strong associations with severe CRS, ICANS, and consumptive CARAC. 26 27 100 101 102 A simplified version (sEASIX), excluding creatinine entirely, has likewise shown predictive potential, although available data remain limited to specific cohorts. 26 99

##### Circulating Endothelial Biomarkers

Specific endothelial biomarkers provide additional mechanistic insight. Retrospective and prospective data demonstrate that endothelial vulnerability often precedes CAR T cell infusion. Compared with healthy controls, patients showed reduced baseline ADAMTS13 activity and elevated VWF antigen, suggesting preexisting endothelial activation or exhaustion characterized by the depletion of intracellular storage pools. 16 87 These baseline abnormalities persisted postinfusion and correlated with toxicity. Patients who later developed CRS grade ≥ 2 exhibited lower baseline ADAMTS13 activity. Similar patterns were observed in ICANS, with reduced ADAMTS13 activity and higher VWF levels compared with patients without neurotoxicity. Notably, higher baseline ADAMTS13 activity was associated with better treatment response, suggesting that the VWF/ADAMTS13 balance may reflect broader endothelial fitness relevant to therapeutic efficacy. 87 Thus, dysregulation of this axis may precede the cytokine surge as an intrinsic vulnerability rather than a secondary inflammatory consequence.

Imbalance within the VWF/ADAMTS13 axis becomes increasingly pronounced during CRS and ICANS. In advanced ICANS, serum VWF levels may rise four- to fivefold above normal, accompanied by elevated IL-8 levels consistent with WPB release. 16 18 Functional assays demonstrate that serum obtained 3–5 days after infusion from patients with early neurotoxicity induces abundant VWF–platelet string formation on cultured endothelial cells. Conversely, in advanced ICANS, string formation paradoxically decreases despite elevated circulating VWF concentrations. This phase coincides with the depletion of high-molecular-weight VWF multimers and a significantly reduced ADAMTS13/VWF ratio (26.4% vs. 35.2%; *p* = 0.007), suggesting impaired proteolytic regulation of VWF at peak toxicity. 18

Alterations in the Ang pathway further track endothelial destabilization across the treatment timeline. Compared with healthy controls, CAR T cell recipients exhibit elevated Ang-2 levels at baseline, which rise further postinfusion. Importantly, increases in Ang-2, the Ang-2/Ang-1 ratio, and soluble endothelial adhesion molecules (sE-selectin, sVCAM-1, sICAM-1) are detectable days before clinical CRS onset, differentiating mild from severe CRS within the first 72 h. 16 17 18 Predictive models incorporating sVCAM-1 and the Ang-2/Ang-1 ratio show high specificity for identifying patients at risk of severe toxicity, 16 17 while early Ang-2 elevation similarly associates with ICANS severity. 18 At peak toxicity, severe CRS and ICANS are consistently characterized by marked increases in Ang-2 and soluble adhesion molecules, alongside a decline in Ang-1 concentrations. This coordinated shift toward an elevated Ang-2/Ang-1 ratio drives a pro-thrombotic endothelial phenotype across various CAR constructs—including CD19- and CD28-based products—with the reduction in Ang-1 likely exacerbated by concurrent thrombocytopenia. 17 19 25 100 These trajectories are summarized in Table 1 .

**Table 1:** Endothelial-related biomarker dynamics during CAR T cell therapy.

Biomarker	Baseline (preinfusion)	Early postinfusion phase	Peak CRS	Peak ICANS	Mechanistic interpretation
VWF antigen	Elevated	Further increase due to WPB exocytosis	Markedly increased	Very high (up to 4–5× normal)	WPB release/marker of endothelial activation
ADAMTS13 activity	Reduced	Further decline	Reduced	Reduced with low ADAMTS13/VWF ratio	Impaired ULVWF cleavage/relative ADAMTS13 insufficiency
Ang-2	Elevated	Increased (associated with severe CRS/ICANS)	Markedly increased	Markedly increased	WPB release/Tie2 antagonism/increased vascular permeability
Ang-1	Normal to mildly reduced	Slight decline	Reduced	Reduced	Loss of vascular stability/reduced Tie2 signaling
Soluble adhesion molecules (sVCAM-1, sICAM-1, sE-selectin)	Variable	Increased (distinguishes mild vs. severe CRS)	High	High	Markers of endothelial activation

Beyond diagnostic correlation, early Ang kinetics offer significant risk-stratification potential. Ang-2 elevations within the first 24 hours postinfusion forecast the subsequent need for intensive care support. A multivariable model integrating Ang-2, soluble ST2, and NET markers showed good predictive accuracy (AUC: 0.80), while a broader biomarker panel including Ang-2, NETs, sC5b-9, VWF, and PAI-1 reliably distinguished severe CAR-related toxicity from sepsis. 16 Elevated endothelial activation markers have similarly been associated with early renal and hepatic dysfunction. 17 Nonetheless, biomarker patterns across studies are not uniformly consistent; a prospective pediatric and young adults cohort found no significant differences in Ang kinetics in relation to ICANS, potentially reflecting age-related biological variation or confounding by concurrent CRS. 103

From a clinical perspective, the utility of endothelial biomarkers appears to depend strongly on timing. Baseline abnormalities may identify patients with preexisting endothelial vulnerability, whereas early postinfusion changes may help recognize individuals at increased risk of severe toxicity. In contrast, peak biomarker elevations largely reflect the severity of established CRS or ICANS and therefore have greater prognostic than predictive value. At present, endothelial biomarkers should be regarded as complementary to clinical risk assessment and composite indices such as EASIX rather than as standalone decision-making tools. Key clinical studies evaluating VWF, ADAMTS13, and Angs in CAR T cell-associated toxicities are summarized in Table 2 .

**Table 2:** Overview of studies investigating VWF, ADAMTS13, and angiopoietins in CAR T cell therapy-related toxicities.

Study	Cohort (disease and CAR T product)	Study design	Primary and secondary endpoints	Key findings
Gust et al (2017) 18	R/R B-ALL, NHL, or CLL adult patients ( *n* = 133) treated with anti-CD19-CAR lentiviral vector-transduced autologous T-cells (41BB-costimulatory domain)	Phase I/II, single-center CAR-T cell dose escalation/de-escalation clinical trial (NCT01865617)	Characterization of neurological AEs (ICANS) within 28 d post-CAR-T cell infusion	• ↑ Ang-2, ↑ VWF and Ang-2/Ang-1 ratio in grade ≥ 4 ICANS vs. grade ≤ 3 • ↓ ADAMTS13 activity and ADAMTS13/VWF ratio in severe cases • ↓ HMW VWF multimers and ↑ LMW VWF multimers in grade ≥ 4 ICANS vs. grade ≤ 3 • Formation of VWF-platelet strings on ECs in vitro
Hay et al (2017) 19	R/R B-ALL, NHL, or CLL adult patients ( *n* = 133) treated with anti-CD19-CAR lentiviral vector-transduced autologous T-cells (41BB-costimulatory domain)	Phase I/II, single-center CAR-T cell dose escalation/de-escalation clinical trial (NCT01865617)	Kinetics and biomarkers of severe CRS	• ↓Ang-1, ↑ VWF, ↑ Ang-2 and ↑ Ang-2/Ang-1 ratio in severe CRS (grade ≥ 4) compared with lower grades
Santomasso et al (2019) 25	R/R B-ALL adult patients ( *n* = 53) treated with 19–28z anti-CD19 autologous CAR T-cells (CD28 costimulatory domain)	Phase I (NCT01044069) single-center clinical trial	Assess safety and efficacy of 19–28z CAR T-cells	• ↓Ang-1 and ↑ Ang-2/Ang-1 ratio in severe ICANS (grade ≥ 3) vs. lower grades • ↑ Ang-2 in severe CRS vs. mild CRS • Lower baseline platelet count correlated with severe ICANS
Gust et al (2019) 103	R/R B-ALL, both pediatric and young adults ( *n* = 43) treated with CD19-directed CAR T-cells (SCRI-CAR19v1; 41BB-costimulatory domain)	Prospective cohort study	Assessed systemic cytokine release, glial injury, and neuroinflammation in CAR T-associated ICANS	• No significant differences in VEGF-A, Ang-1, or Ang-2 between patients with and without ICANS
Hong et al (2021) 17	R/R B-ALL, both pediatric and adult patients ( *n* = 30) treated with humanized CD19-targeted CAR T-cells (hCART19s) vs. healthy controls ( *n* = 7)	Prospective cohort study	Assessment of endothelial cell activation biomarkers in relation to the occurrence and severity of CRS	• ↓Ang-1, ↑ VWF, ↑ Ang-2 and ↑ Ang2/Ang-1 ratio in severe CRS (grade ≥ 4) vs. lower grades • Peak VWF and Ang-2 were higher at CRS peak than at onset; VWF, Ang-1, Ang-2 and Ang-2/Ang-1 ratio declined upon CRS recovery • Endothelial activation correlated with hepatic, renal and hematopoietic dysfunction
Galli et al (2022) 100	Aggressive lymphoproliferative disorders in adults ( *n* = 38) treated with anti-CD19 CAR T-cells (tisagenlecleucel, axicabtagene ciloleucel, and brexucabtagene autoleucel)	Prospective cohort study	Assessment of the impact and interrelation of hypercoagulation, acute toxicities, and endotheliopathy (measured by mEASIX) in the early post-CAR-T phase	• CRS grade ≥ 2: ↑ PT, aPTT, fibrinogen, D-dimer, FVIII, VWF and ↓ platelet count and antithrombin levels • ICANS: ↑ PT, D-dimer, FVIII, and VWF levels and ↓ fibrinogen and platelet counts • A higher mEASIX score correlated with ↑ aPTT, fibrinogen, D-dimer, FVIII, and VWF and ↓ antithrombin levels
Moreno-Castano et al (2023) 16	R/R B-ALL and NHL adult patients ( *n* = 62) treated with anti-CD19 CAR T-cells (varnimcabtagene autoleucel, tisagenlecleucel, axicabtagene ciloleucel, lisocabtagene maraleucel), and academic CAR T-cells against BCMA for MM (ARI0002h) vs. healthy controls ( *n* = 49)	Prospective cohort study	Assessment of endotheliopathy, innate immune activation, and hemostatic alterations in CAR-T cell toxicity; comparison with sepsis for differential diagnosis	• All biomarkers were significantly increased in patients with CRS/ICANS vs. the control group • Higher ADAMTS13 activity in the control group • Ang-2 and NETs: Identified as potential early predictors of CAR-T cell toxicity onset and severity • Ang-2, NETs, sC5b-9, VWF:Ag and PAI-1 Ag effectively distinguished severe CAR-T cell toxicities from sepsis
Jess et al (2023) 114	R/R B-ALL, both pediatric and young adults ( *n* = 62) treated with anti-CD22 CAR T-cells (41BB-costimulatory domain)	Retrospective cohort study	Characterization of hematologic toxicities and coagulopathy associated with CRS, including correlations with ICANS and HLH-like toxicity	• ↑ Ang-2 and Ang-2/Ang-1 ratio in patients with ICANS (days 10–14 postinfusion) • No differences in protein C, protein S, factor VIII, antithrombin, VWF:Ag, or Ang-1 between patients with and without coagulopathy
Shoaib et al (2025) 87	R/R DLBCL adult patients ( *n* = 62) treated with anti-CD19 CAR T-cells (axicabtagene ciloleucel, tisagenlecleucel and others) vs. healthy controls ( *n* = 30)	Retrospective longitudinal cohort study	Assessment of plasma ADAMTS13 activity, VWF:Ag and syndecan-1 before and after CAR-T infusion; association with response and toxicity	• Preinfusion ADAMTS13 reduced; VWF elevated (endothelial priming) • Trend toward lower baseline ADAMTS13 in CRS ≥ II • Lower ADAMTS13/higher VWF in ICANS • Higher baseline ADAMTS13 associated with better treatment response

Abbreviations: ADAMTS13, a disintegrin-like and metalloprotease with thrombospondin type 1 repeats; AEs, adverse events; Ang, angiopoietin; aPTT, activated partial thromboplastin time; CLL, chronic lymphocytic leukemia; CRS, cytokine release syndrome; DIC, disseminated intravascular coagulation; DLBCL, diffuse large B cell lymphoma; ECs, endothelial cells; HLH, hemophagocytic lymphohistiocytosis; HMW, high molecular weight; ICANS, Immune effector cell-associated neurotoxicity syndrome; LMW, low molecular weight; mEASIX, modified version of endothelial activation and stress index; MM, multiple myeloma; NET, neutrophil extracellular traps; NHL, non-Hodgkin lymphoma; PAI-1 Ag, plasminogen activator inhibitor-1 antigen; PT, prothrombin time; R/R, relapsed/refractory; sC5b-9, soluble terminal fraction of the complement system membrane attack complex; VEGF, vascular endothelial growth factor; VWF:Ag, von Willebrand Factor antigen.

##### Sources of Heterogeneity and Methodological Limitations

When interpreting these biomarker trajectories, several sources of clinical and biological heterogeneity should be considered. First, costimulatory domains influence kinetics: CD28-costimulated constructs (e.g., axicabtagene ciloleucel) frequently exhibit more rapid in vivo expansion and earlier cytokine peaks than 4–1BB-based products (e.g., tisagenlecleucel), driving earlier CRS onset and higher rates of neurotoxicity. 104 105 106 Second, discordant findings between adult and pediatric cohorts suggest that age-related differences and baseline vascular fitness may influence endothelial responses. Adult patients often present with extensive prior treatment exposure, vascular comorbidities, and preexisting neurovascular risk factors, whereas pediatric patients may possess a more resilient endothelial baseline. Consistent with this concept, variations in endothelial biomarkers have been detected before lymphodepletion and at the time of infusion. 18 81 Finally, the contribution of lymphodepleting chemotherapy should be considered. Fludarabine- and cyclophosphamide-based conditioning may itself induce endothelial injury and perturbations in the VWF/ADAMTS13 axis prior to CAR T cell expansion. Consequently, early biomarker abnormalities may partly reflect chemotherapy-induced vascular stress rather than exclusively CAR T cell-mediated toxicity. 12 13 18 81

Compounding these biological differences, current evidence remains limited by relatively small cohorts and substantial methodological variability. Assays for VWF and Angs lack cross-platform standardization. Studies variably report VWF antigen levels without assessing functional activity or multimer distribution, potentially obscuring clinically relevant qualitative differences. Similarly, Ang-1 measurements are highly sensitive to preanalytical conditions, particularly platelet activation during sample collection. Importantly, clinically validated thresholds remain unavailable, precluding routine biomarker-guided decision-making. Consequently, these markers should currently be regarded as candidate biomarkers and pathophysiological correlates rather than established clinical tools.

In conclusion, current evidence supports a model in which endothelial injury acts as a potential bridge linking CAR T cell–mediated immune hyperactivation to vascular leak, dysregulated hemostasis, and neurovascular injury. Despite biological and methodological heterogeneity across studies, observed perturbations in the VWF/ADAMTS13 axis, the Ang-2/Ang-1 balance, and endothelial adhesion molecules frequently correlate with toxicity severity, lending support to the concept of CRS and ICANS as therapy-related endotheliopathies.

### CAR T Cell Therapy-related Coagulopathy: A Consequence of Endotheliopathy

Coagulation abnormalities following CAR T cell infusion occur in the context of systemic inflammation and are best interpreted as the hematologic expression of cytokine-driven vascular injury rather than a primary coagulation disorder. Laboratory-defined coagulopathy has been reported in up to 60% of treated patients, with the highest incidence observed in multiple myeloma cohorts receiving BCMA-directed products. Risk factors include high tumor burden, extensive prior therapy, low baseline platelet counts, and, most prominently, the presence and severity of CRS. 107 108 109 110 111

These abnormalities evolve in parallel with the inflammatory cascade characteristic of CRS. Rising IL-6, CRP, and IFN-γ levels coincide with increasing D-dimer concentrations, prolongation of PT and aPTT, and enhanced thrombin generation. Platelet counts and fibrinogen levels decline proportionally to CRS severity, with hypofibrinogenemia becoming particularly pronounced in severe CRS or immune effector cell–associated hemophagocytic syndrome (IEC-HS), where it may serve as a diagnostic criterion. 88 107 108 109 110 112 113 114 Consistent with this inflammatory–hemostatic interplay, elevated mEASIX scores during CRS grade ≥ 2 correlate with increased VWF antigen, FVIII, and D-dimer levels, together with PT/aPTT prolongation, underscoring the convergence of vascular injury and hemostatic stress. 100

Clinically, CARAC typically begins around day 4 postinfusion, peaks between days 8 and 9, and gradually resolves as cytokine levels normalize. The steep fall in fibrinogen during peak CRS, occasionally accentuated after IL-6 receptor blockade with tocilizumab, illustrates the tight coupling between inflammatory signaling and hepatic acute-phase regulation. 25 107 108 109 110 112 115 116

Mechanistically, inflammatory cytokines promote a procoagulant state characterized by enhanced thrombin generation, impaired fibrinolysis, and consumption of platelets and clotting factors. Suppression of endogenous anticoagulant pathways (antithrombin, protein C, protein S), together with elevated PAI-1, further destabilizes hemostatic balance. The resulting phenotype resembles a consumptive coagulopathy with TMA–like features, typified by thrombocytopenia, clotting factor depletion, fibrinogen consumption, and secondary fibrinolysis. 12 107 108 109 110 112 113 115 116 117 118 119 120 Supporting the link between endothelial injury and hemostatic derangements, patients who developed CARAC in a phase I anti-CD22 CAR T cell study exhibited significantly higher Ang-2 levels and Ang-2/Ang-1 ratios. 114 The principal laboratory abnormalities observed during CARAC are summarized in Table 3 .

**Table 3:** Key laboratory abnormalities observed in CAR T cell therapy–associated coagulopathy (CARAC).

Parameter	Typical change during CARAC	Approximate magnitude ^a^	Mechanistic basis	Clinical implication
Platelet count	↓	Often < 50 × 10 ^9^ /L in severe CARAC; may fall below 20 × 10 ^9^ /L in DIC-like presentations	Consumption during thrombo-inflammation	Bleeding risk
Fibrinogen	↓	Often < 1.5 g/L in severe CRS; may fall below 1.0 g/L in IEC-HS/DIC-like states	Consumptive coagulopathy; impaired hepatic synthesis	Marker of severe CRS/IEC-HS
D-dimer	↑	Frequently > 5 mg/L; may exceed 20 mg/L in severe coagulopathy	Increased fibrin formation and fibrinolysis	Indicator of thrombo-inflammation
PT	Prolonged	Typically 15–20 s, >20 s in severe cases	Coagulation factor consumption	Reflects evolving coagulopathy
aPTT	Prolonged	Typically 40–60 s; may exceed 90 s in severe cases	Coagulation factor consumption	Reflects evolving coagulopathy
Antithrombin	↓	Variable reduction	Consumption and reduced synthesis	Loss of anticoagulant control
Protein C/S	↓	Variable reduction	Inflammatory suppression	Reduced anticoagulant capacity

^a^ Approximate magnitudes are derived from heterogeneous studies and consensus criteria and should not be interpreted as validated clinical thresholds.

In severe cases, the laboratory profile may approach criteria for disseminated intravascular coagulation (DIC), although fulminant DIC remains uncommon. Parallels with macrophage activation syndrome and hemophagocytic lymphohistiocytosis suggest that hypofibrinogenemia during CRS may partly reflect primary hyperfibrinolysis, potentially driven by macrophage-derived plasminogen and cytokines such as IL-1β and TNF-α. 111 112 These insights position CARAC within a broader continuum of inflammation-mediated thrombo-inflammatory syndromes rather than as a discrete coagulation disorder.

Despite frequent laboratory abnormalities, overt thrombotic and bleeding events affect a minority of patients but carry significant clinical weight. A comprehensive narrative review highlighted that while VTE rates remain low in highly selected trial populations (0.48–3.26%), real-world observational cohorts report substantially higher VTE rates ranging from 2.1% to 10.8%. This discrepancy is mirrored in bleeding complications, which range from 0% to 1.85% in clinical trials up to 2.8% to 12.5% in real-world registries, with gastrointestinal and mucosal sites predominating. 111 113 117 121 122 123 Further quantifying this risk, a large-scale meta-analysis of 24 studies encompassing nearly 3000 patients established a pooled VTE proportion of 6.16% (adjusting up to 7.82% after accounting for publication bias), with most events clustering within the first 50 days postinfusion. Crucially, this analysis confirmed that severe immune toxicities dramatically compound this risk, with grade >2 CRS (RR = 4.12) and ICANS (RR = 3.43) serving as powerful independent predictors of VTE. 124

These systematic findings align closely with a recent multicenter Canadian cohort of 561 CD19 CAR T recipients that reported a 90-day cumulative incidence of 6.7% for VTE and 6.5% for clinically relevant bleeding. While no fatal pulmonary embolism occurred, two fatal intracranial hemorrhages were observed in thrombocytopenic patients not receiving thromboprophylaxis. 125 These findings demonstrate that thrombotic and hemorrhagic complications, although affecting a minority of patients, are clinically meaningful and closely linked to severe CRS, multiorgan dysfunction, and the need for intensive care support. Nevertheless, granular prospective data directly mapping the real-time kinetics of specific coagulation and fibrinolysis biomarkers alongside clinical thrombotic events remain scarce in current literature.

Compounding this scarcity of data, terminology in this field remains inconsistent. The term CARAC has been used to describe a spectrum ranging from isolated laboratory abnormalities to TMA-like presentations and, rarely, overt DIC. Such heterogeneity complicates comparison across studies and impedes clinical interpretation. Recent commentaries therefore emphasize the need for standardized definitions and grading systems, analogous to those used for CRS and ICANS, to distinguish transient endothelial activation from clinically significant consumptive coagulopathy. 111 126 Addressing this need, recently proposed consensus criteria by Schoettler et al define TMA in the setting of cellular therapies as the presence of microangiopathic hemolytic anemia (characterized by schistocytes and elevated LDH), persistent or consumptive thrombocytopenia, and concurrent renal or neurological dysfunction, often accompanied by markers of terminal complement activation such as elevated soluble C5b-9. 127 This distinction is clinically important, as true TMA may require targeted intervention beyond standard supportive care.

Overall, current data support the concept that CARAC represents the hematologic correlate of cytokine-mediated endothelial injury. Integrating this vascular perspective into clinical practice may improve risk stratification and guide interventions aimed at maintaining endothelial stability.

## Clinical Implications and Future Perspectives

Recognition of endotheliopathy as a central driver of CRS, ICANS, and CARAC has prompted interest in vascular-targeted interventions. Yet, despite growing mechanistic evidence supporting a central role for endothelial injury in CAR T cell–related toxicities, no endothelial-specific therapy is currently approved in this setting. At present, management remains centered on supportive care and control of the underlying inflammatory response, highlighting a substantial gap between preclinical mechanistic understanding and clinical translation.

### Established Therapeutic Approaches

Interventions for CAR T cell–related toxicities primarily target the inflammatory cascade driving endotheliopathy. Supportive care, corticosteroids, and IL-6 receptor blockade with tocilizumab remain the cornerstones of CRS management and have substantially improved clinical outcomes. 7 9 76 81 Although these interventions do not directly target the endothelium, their efficacy indirectly underscores the central contribution of vascular injury to the pathogenesis of CRS and related complications. 89 128 The resolution of thrombotic and bleeding complications after CAR T cell therapy follows established hematological principles but should be individualized according to the evolving balance between thrombosis risk, thrombocytopenia, and coagulopathy, particularly during the first weeks after infusion. 113 116 123

### Investigational Endothelial-Protective Strategies

Several endothelial-protective approaches have been proposed and are supported by preclinical, translational, or early clinical evidence. Agents with immunomodulatory and endothelial-stabilizing properties, including statins and defibrotide, have attracted particular interest because of their established role in other endothelial injury syndromes. Statins reduce pro-inflammatory cytokine signaling and leukocyte adhesion, whereas defibrotide promotes endothelial protection through increased NO bioavailability and reduced oxidative stress. Though neither strategy has been systematically evaluated in CAR T cell recipients, both represent promising options for mitigating therapy-related endotheliopathy. 12 129 130 Likewise, targeting upstream inflammatory mediators may indirectly attenuate vascular injury. Given the central role of IL-1β and TNF-α in driving endotheliopathy during CAR T cell therapy, cytokine-directed approaches have emerged as compelling therapeutic strategies. Among these, IL-1 blockade with anakinra has gained increasing clinical acceptance for the management of refractory CRS, ICANS, and IEC-HS, 89 90 whereas TNF-α inhibition with agents such as adalimumab remains at an earlier stage of clinical development. Early clinical and translational studies suggest that these approaches may limit therapy-related vascular injury without compromising antitumor efficacy. 81 131 132 Nevertheless, clinical experience remains limited, and prospective studies are required before endothelial-targeted cytokine blockade can be safely incorporated into routine practice.

### Experimental Pathway-Directed Approaches

Beyond general endothelial protection, several therapeutic concepts seek to directly modulate the endothelial pathways discussed in this review. Currently, these strategies remain strictly experimental and are mainly extrapolated from thrombotic microangiopathies, sepsis, and other forms of systemic endothelial injury. Within the VWF/ADAMTS13 axis, potential interventions include recombinant ADAMTS13 administration to cleave excessive ULVWF multimers, 133 and VWF-directed therapies such as caplacizumab to reduce pathological platelet–VWF interactions. 134 Recombinant ADAMTS13 has been shown ex vivo to normalize VWF multimer distribution in plasma obtained from patients with severe COVID-19, providing proof-of-concept that restoration of the VWF/ADAMTS13 balance may be therapeutically feasible in inflammatory endotheliopathies. 135 However, translating these anti-VWF strategies into the CAR T cell arena poses significant clinical challenges. The use of caplacizumab, an anti-VWF nanobody, carries a profound risk of inducing severe, life-threatening hemorrhage. 134 This risk is exponentially amplified in CAR T cell recipients, who frequently experience deep, prolonged cytopenias and consumptive coagulopathies. 113 116 Consequently, defining whether the benefit of preventing microvascular thrombosis outweighs the substantial bleeding risk remains a critical, unanswered question that has yet to be evaluated in prospective trials.

Another emerging avenue centers on modulation of the Ang-Tie2 axis. Experimental studies consistently show that enhancing Tie2 signaling stabilizes endothelial junctions, reduces vascular leakage, and improves survival in inflammatory injury models. Gene-based Ang-1 delivery has improved vascular integrity in sepsis and organ injury models. 136 137 138 139 Vasculotide, a synthetic Tie2 agonist, reduces vascular permeability and mortality across multiple preclinical inflammatory settings, 136 140 141 whereas the Ang-1 mimetic MAT.Ang-1 similarly decreases vascular leakage without hemodynamic compromise. 142 Conversely, Ang-2 inhibition reduces vascular leak and organ injury; notably, combining Ang-2 blockade and Tie2 activation appears more effective than either strategy alone in preserving glycocalyx integrity and improving survival. 61 67 136 Although these findings provide compelling proof-of-concept for endothelial-targeted interventions, their applicability to CAR T cell–associated toxicities remains entirely untested. The principal endothelial-targeted therapeutic approaches discussed in this review, together with their targets and current level of evidence, are summarized in Table 4 .

**Table 4:** Endothelial-targeted therapeutic strategies in CAR T cell–associated toxicities.

Therapeutic approach	Target pathway	Current evidence
Tocilizumab	IL-6 signaling	Established standard of care/guideline-supported
Corticosteroids	Broad inflammatory pathways	Established standard of care/guideline-supported
Anakinra	IL-1 signaling	Guideline-supported salvage therapy for refractory toxicities
TNF-α inhibitors (e.g., adalimumab)	TNF-α signaling	Preclinical and very limited clinical experience (case-based)
Defibrotide	Endothelial protection	Limited clinical experience/under investigation
Statins	Endothelial stabilization	Preclinical and observational evidence
Recombinant ADAMTS13	VWF/ADAMTS13 axis	Preclinical and mechanistic rationale/no clinical studies in CAR T cell therapy
Caplacizumab	VWF-mediated platelet adhesion	Extrapolated from TTP/no clinical studies in CAR T cell therapy
Tie2 agonists (e.g., vasculotide)	Ang–Tie2 pathway	Preclinical evidence
Ang-2 blockade	Ang–Tie2 pathway	Preclinical evidence

### Future Directions

Future research priorities include prospective multicenter validation of endothelial candidate biomarkers such as the VWF/ADAMTS13 ratio, ADAMTS13 activity, and Ang profiles, as well as the establishment of clinically actionable thresholds for risk stratification. Importantly, a distinction should be made between biomarkers that identify patients at increased risk of toxicity before treatment and those that reflect evolving toxicity severity after CAR T cell infusion. Baseline assessment of endothelial status prior to lymphodepletion may help identify patients with preexisting endothelial vulnerability, whereas dynamic biomarker changes during therapy may provide prognostic information regarding disease trajectory and treatment response. Ultimately, clinical trials integrating biomarker-guided stratification with mechanistically targeted interventions will be required to determine whether modulation of the VWF/ADAMTS13 axis or Ang–Tie2 pathway can safely alter toxicity trajectories without compromising antitumor efficacy.

## Conclusion

CAR T cell therapy has fundamentally reshaped the treatment landscape of hematologic malignancies, yet its clinical impact is increasingly shaped by vascular toxicities driven by endothelial injury and dysregulated hemostasis. Growing evidence identifies cytokine-driven endotheliopathy as a unifying mechanism linking immune hyperactivation to immunothrombosis, vascular instability, and organ dysfunction, mediated in part through perturbations of the VWF/ADAMTS13 axis and the Ang-2–Tie2 signaling pathway.

Reframing CAR T cell–related toxicities as manifestations of underlying endothelial vulnerability provides a coherent biological framework and highlights opportunities for earlier risk stratification. Endothelial biomarkers may help identify patients at increased risk of toxicity and improve understanding of the mechanisms underlying CRS, ICANS, and CARAC. However, a major translational challenge remains the absence of standardized assays, validated thresholds, and external prospective validation.

Future studies should focus on defining the clinical utility of baseline endothelial assessment and early postinfusion biomarker monitoring, while integrating these approaches into prospective risk-stratification strategies. Although these biomarkers remain investigational, continued refinement of endothelial profiling may ultimately contribute to safer and more personalized CAR T cell therapy.

## References

[1] Dabas P Danda A Revolutionizing cancer treatment: a comprehensive review of CAR-T cell therapy Med Oncol 2023 40 09 275 37608202 10.1007/s12032-023-02146-y

[2] Zhang X Zhu L Zhang H Chen S Xiao Y CAR-T cell therapy in hematological malignancies: current opportunities and challenges Front Immunol 2022 13 927153 35757715 10.3389/fimmu.2022.927153 PMC9226391

[3] Pasqui D M Latorraca C DOC Pacheco R L Riera R CAR-T cell therapy for patients with hematological malignancies. A systematic review Eur J Haematol 2022 109 06 601 618 36018500 10.1111/ejh.13851

[4] Roex G Feys T Beguin Y Chimeric antigen receptor-T-cell therapy for B-cell hematological malignancies: an update of the pivotal clinical trial data Pharmaceutics 2020 12 02 194 32102267 10.3390/pharmaceutics12020194 PMC7076393

[5] Lee D W Santomasso B D Locke F L ASTCT consensus grading for cytokine release syndrome and neurologic toxicity associated with immune effector cells Biol Blood Marrow Transplant 2019 25 04 625 638 30592986 10.1016/j.bbmt.2018.12.758 PMC12180426

[6] Yang C Nguyen J Yen Y Complete spectrum of adverse events associated with chimeric antigen receptor (CAR)-T cell therapies J Biomed Sci 2023 30 01 89 37864230 10.1186/s12929-023-00982-8 PMC10590030

[7] Ferreri C J Bhutani M Mechanisms and management of CAR T toxicity Front Oncol 2024 14 1.39649E6 10.3389/fonc.2024.1396490 PMC11148294 38835382

[8] Kim S J Yoon S E Kim W S Current challenges in chimeric antigen receptor T-cell therapy in patients with B-cell lymphoid malignancies Ann Lab Med 2024 44 03 210 221 38205527 10.3343/alm.2023.0388 PMC10813822

[9] Li Y Ming Y Fu R The pathogenesis, diagnosis, prevention, and treatment of CAR-T cell therapy-related adverse reactions Front Pharmacol 2022 13 950923 36313336 10.3389/fphar.2022.950923 PMC9616161

[10] Levstek L Janžič L Ihan A Kopitar A N Biomarkers for prediction of CAR T therapy outcomes: current and future perspectives Front Immunol 2024 15 1.378944E6 10.3389/fimmu.2024.1378944 PMC10979304 38558801

[11] Cordas Dos Santos D M Tix T Shouval R A systematic review and meta-analysis of nonrelapse mortality after CAR T cell therapy Nat Med 2024 30 09 2667 2678 38977912 10.1038/s41591-024-03084-6 PMC11765209

[12] Moreno-Castaño A B Iraola G Martínez-Cibrián N Endothelial dysfunction and hemostatic imbalance in CAR T-cell-associated toxicities: pathophysiological insights and the role of circulating biomarkers Front Immunol 2025 16 1.699894E6 10.3389/fimmu.2025.1699894 PMC12620366 41256862

[13] Gavriilaki E Sakellari I Gavriilaki M Anagnostopoulos A A new era in endothelial injury syndromes: toxicity of CAR-T cells and the role of immunity Int J Mol Sci 2020 21 11 3886 32485958 10.3390/ijms21113886 PMC7312228

[14] Mackall C L Miklos D B CNS endothelial cell activation emerges as a driver of CAR T cell-associated neurotoxicity Cancer Discov 2017 7 12 1371 1373 29208775 10.1158/2159-8290.CD-17-1084

[15] Wang Z Han W Biomarkers of cytokine release syndrome and neurotoxicity related to CAR-T cell therapy Biomark Res 2018 6 4 29387417 10.1186/s40364-018-0116-0 PMC5778792

[16] Moreno-Castaño A B Fernández S Ventosa H Characterization of the endotheliopathy, innate-immune activation and hemostatic imbalance underlying CAR-T cell toxicities: laboratory tools for an early and differential diagnosis J Immunother Cancer 2023 11 04 e006365 37045474 10.1136/jitc-2022-006365 PMC10106034

[17] Hong F Shi M Cao J Predictive role of endothelial cell activation in cytokine release syndrome after chimeric antigen receptor T cell therapy for acute lymphoblastic leukaemia J Cell Mol Med 2021 25 24 11063 11074 34734474 10.1111/jcmm.17029 PMC8650023

[18] Gust J Hay K A Hanafi L A Endothelial activation and blood-brain barrier disruption in neurotoxicity after adoptive immunotherapy with CD19 CAR-T cells Cancer Discov 2017 7 12 1404 1419 29025771 10.1158/2159-8290.CD-17-0698 PMC5718945

[19] Hay K A Hanafi L A Li D Kinetics and biomarkers of severe cytokine release syndrome after CD19 chimeric antigen receptor-modified T-cell therapy Blood 2017 130 21 2295 2306 28924019 10.1182/blood-2017-06-793141 PMC5701525

[20] Delvaeye M Conway E M Coagulation and innate immune responses: can we view them separately? Blood 2009 114 12 2367 2374 19584396 10.1182/blood-2009-05-199208

[21] Gaertner F Massberg S Blood coagulation in immunothrombosis-at the frontline of intravascular immunity Semin Immunol 2016 28 06 561 569 27866916 10.1016/j.smim.2016.10.010

[22] Jackson S P Darbousset R Schoenwaelder S M Thromboinflammation: challenges of therapeutically targeting coagulation and other host defense mechanisms Blood 2019 133 09 906 918 30642917 10.1182/blood-2018-11-882993

[23] Marcos-Jubilar M Lecumberri R Páramo J A Immunothrombosis: molecular aspects and new therapeutic perspectives J Clin Med 2023 12 04 1399 36835934 10.3390/jcm12041399 PMC9958829

[24] Schrottmaier W C Assinger A The concept of thromboinflammation Hamostaseologie 2024 44 01 21 30 38417802 10.1055/a-2178-6491 PMC12997454

[25] Santomasso B D Park J H Salloum D Clinical and biological correlates of neurotoxicity associated with CAR T-cell therapy in patients with B-cell acute lymphoblastic leukemia Cancer Discov 2018 8 08 958 971 29880584 10.1158/2159-8290.CD-17-1319 PMC6385599

[26] de Boer J W Keijzer K Pennings E RA Population-based external validation of the EASIX scores to predict CAR T-cell-related toxicities Cancers 2023 15 22 5443 38001703 10.3390/cancers15225443 PMC10670876

[27] Korell F Penack O Mattie M EASIX and severe endothelial complications after CD19-directed CAR-T cell therapy-a cohort study Front Immunol 2022 13 877477 35464403 10.3389/fimmu.2022.877477 PMC9033201

[28] Frenking J H Zhou X Wagner V EASIX-guided risk stratification for complications and outcome after CAR T-cell therapy with ide-cel in relapsed/refractory multiple myeloma J Immunother Cancer 2024 12 10 e009220 39379098 10.1136/jitc-2024-009220 PMC11459298

[29] Aird W C Spatial and temporal dynamics of the endothelium J Thromb Haemost 2005 3 07 1392 1406 15892866 10.1111/j.1538-7836.2005.01328.x

[30] Esmon C T Coagulation inhibitors in inflammation Biochem Soc Trans 2005 33(Pt 2)401 405 15787615 10.1042/BST0330401

[31] Jin R C Voetsch B Loscalzo J Endogenous mechanisms of inhibition of platelet function Microcirculation 2005 12 03 247 258 15814434 10.1080/10739680590925493

[32] van Hinsbergh V W Endothelium--role in regulation of coagulation and inflammation Semin Immunopathol 2012 34 01 93 106 21845431 10.1007/s00281-011-0285-5 PMC3233666

[33] De Nardi A C Coy-Canguçu A Saito A Immunothrombosis and its underlying biological mechanisms Hematol Transfus Cell Ther 2024 46 01 49 57 37451977 10.1016/j.htct.2023.05.008 PMC10935458

[34] Bernardo A Ball C Nolasco L Moake J F Dong J F Effects of inflammatory cytokines on the release and cleavage of the endothelial cell-derived ultralarge von Willebrand factor multimers under flow Blood 2004 104 01 100 106 15026315 10.1182/blood-2004-01-0107

[35] Wang J Doran J The many faces of cytokine release syndrome-related coagulopathy Clin Hematol Int 2021 3 01 3 12 34595461 10.2991/chi.k.210117.001 PMC8432322

[36] Engelmann B Massberg S Thrombosis as an intravascular effector of innate immunity Nat Rev Immunol 2013 13 01 34 45 23222502 10.1038/nri3345

[37] Páramo J A Lecumberri R New mechanisms in vein thrombosis: Immunothrombosis Med Clin (Barc) 2019 153 02 78 81 30803800 10.1016/j.medcli.2019.01.001

[38] Stark K Massberg S Interplay between inflammation and thrombosis in cardiovascular pathology Nat Rev Cardiol 2021 18 09 666 682 33958774 10.1038/s41569-021-00552-1 PMC8100938

[39] Maneta E Aivalioti E Tual-Chalot S Endothelial dysfunction and immunothrombosis in sepsis Front Immunol 2023 14 1.144229E6 10.3389/fimmu.2023.1144229 PMC10110956 37081895

[40] Bonaventura A Vecchié A Dagna L Endothelial dysfunction and immunothrombosis as key pathogenic mechanisms in COVID-19 Nat Rev Immunol 2021 21 05 319 329 33824483 10.1038/s41577-021-00536-9 PMC8023349

[41] Fogarty H Ward S E Townsend L Sustained VWF-ADAMTS-13 axis imbalance and endotheliopathy in long COVID syndrome is related to immune dysfunction J Thromb Haemost 2022 20 10 2429 2438 35875995 10.1111/jth.15830 PMC9349977

[42] Cortes G A Moore M J El-Nakeep S Physiology, Von Willebrand Factor Treasure Island (FL) StatPearls 2025 32644488

[43] Leebeek F W Eikenboom J C Von Willebrand’s disease N Engl J Med 2016 375 21 2067 2080 27959741 10.1056/NEJMra1601561

[44] Seidizadeh O Eikenboom J CJ Denis C V von Willebrand disease Nat Rev Dis Primers 2024 10 01 51 39054329 10.1038/s41572-024-00536-8

[45] Atiq F O’Donnell J S Novel functions for von Willebrand factor Blood 2024 144 12 1247 1256 38728426 10.1182/blood.2023021915 PMC11561537

[46] DeYoung V Singh K Kretz C A Mechanisms of ADAMTS13 regulation J Thromb Haemost 2022 20 12 2722 2732 36074019 10.1111/jth.15873 PMC9826392

[47] Crawley J TB de Groot R Xiang Y Luken B M Lane D A Unraveling the scissile bond: how ADAMTS13 recognizes and cleaves von Willebrand factor Blood 2011 118 12 3212 3221 21715306 10.1182/blood-2011-02-306597 PMC3179391

[48] Bockmeyer C L Claus R A Budde U Inflammation-associated ADAMTS13 deficiency promotes formation of ultra-large von Willebrand factor Haematologica 2008 93 01 137 140 18166799 10.3324/haematol.11677

[49] Claus R A Bockmeyer C L Budde U Variations in the ratio between von Willebrand factor and its cleaving protease during systemic inflammation and association with severity and prognosis of organ failure Thromb Haemost 2009 101 02 239 247 19190805

[50] Chen J Chung D W Inflammation, von Willebrand factor, and ADAMTS13 Blood 2018 132 02 141 147 29866815 10.1182/blood-2018-02-769000 PMC6043979

[51] Scully M Cataland S Coppo P Consensus on the standardization of terminology in thrombotic thrombocytopenic purpura and related thrombotic microangiopathies J Thromb Haemost 2017 15 02 312 322 27868334 10.1111/jth.13571

[52] Madarati H Singh K Sparring T Reviewing the dysregulation of Adamts13 and VWF in sepsis Shock 2024 61 02 189 196 38150358 10.1097/SHK.0000000000002291

[53] Peetermans M Meyers S Liesenborghs L Von Willebrand factor and ADAMTS13 impact on the outcome of Staphylococcus aureus sepsis J Thromb Haemost 2020 18 03 722 731 31758651 10.1111/jth.14686

[54] Wibowo A Pranata R Lim M A Akbara M R Martha J W Endotheliopathy marked by high von Willebrand factor (VWF) antigen in COVID-19 is associated with poor outcome: a systematic review and meta-analysis Int J Infect Dis 2022 117 267 273 34192577 10.1016/j.ijid.2021.06.051 PMC8236128

[55] Dachy G Vankeerbergen M Vanlangendonck N Von Willebrand factor as a potential predictive biomarker of early complications of endothelial origin after allogeneic hematopoietic stem cell transplantation Bone Marrow Transplant 2024 59 06 890 892 38459172 10.1038/s41409-024-02242-1

[56] Yang J Wu Z Long Q Insights into immunothrombosis: the interplay among neutrophil extracellular trap, von Willebrand factor, and ADAMTS13 Front Immunol 2020 11 610696 33343584 10.3389/fimmu.2020.610696 PMC7738460

[57] Gragnano F Sperlongano S Golia E The role of von Willebrand factor in vascular inflammation: from pathogenesis to targeted therapy Mediat Inflamm 2017 2017 5.620314E6 10.1155/2017/5620314 PMC5467347 28634421

[58] Grässle S Huck V Pappelbaum K I von Willebrand factor directly interacts with DNA from neutrophil extracellular traps Arterioscler Thromb Vasc Biol 2014 34 07 1382 1389 24790143 10.1161/ATVBAHA.113.303016

[59] Knöbl P NET untangled in TMA? J Thromb Haemost 2015 13 05 729 731 25773706 10.1111/jth.12895

[60] Fiedler U Scharpfenecker M Koidl S The Tie-2 ligand angiopoietin-2 is stored in and rapidly released upon stimulation from endothelial cell Weibel-Palade bodies Blood 2004 103 11 4150 4156 14976056 10.1182/blood-2003-10-3685

[61] Saharinen P Eklund L Alitalo K Therapeutic targeting of the angiopoietin-TIE pathway Nat Rev Drug Discov 2017 16 09 635 661 28529319 10.1038/nrd.2016.278

[62] Page A V Liles W C Biomarkers of endothelial activation/dysfunction in infectious diseases Virulence 2013 4 06 507 516 23669075 10.4161/viru.24530 PMC5359744

[63] Thurston G Daly C The complex role of angiopoietin-2 in the angiopoietin-tie signaling pathway Cold Spring Harb Perspect Med 2012 2 09 a006550 22951441 10.1101/cshperspect.a006650 PMC3426817

[64] Dumont D J Gradwohl G Fong G H Dominant-negative and targeted null mutations in the endothelial receptor tyrosine kinase, tek, reveal a critical role in vasculogenesis of the embryo Genes Dev 1994 8 16 1897 1909 7958865 10.1101/gad.8.16.1897

[65] Sato T N Tozawa Y Deutsch U Distinct roles of the receptor tyrosine kinases Tie-1 and Tie-2 in blood vessel formation Nature 1995 376 6535 70 74 7596437 10.1038/376070a0

[66] Parikh S M Mammoto T Schultz A Excess circulating angiopoietin-2 may contribute to pulmonary vascular leak in sepsis in humans PLoS Med 2006 3 03 e46 16417407 10.1371/journal.pmed.0030046 PMC1334221

[67] David S Mukherjee A Ghosh C C Angiopoietin-2 may contribute to multiple organ dysfunction and death in sepsis ^*^ Crit Care Med 2012 40 11 3034 3041 22890252 10.1097/CCM.0b013e31825fdc31 PMC3705559

[68] van der Heijden M Pickkers P van Nieuw Amerongen G P Circulating angiopoietin-2 levels in the course of septic shock: relation with fluid balance, pulmonary dysfunction and mortality Intensive Care Med 2009 35 09 1567 1574 19551369 10.1007/s00134-009-1560-y PMC2726915

[69] Randi A M Smith K E Castaman G von Willebrand factor regulation of blood vessel formation Blood 2018 132 02 132 140 29866817 10.1182/blood-2018-01-769018 PMC6182264

[70] Groeneveld D J Sanders Y V Adelmeijer J Circulating angiogenic mediators in patients with moderate and severe von Willebrand disease: a multicentre cross-sectional study Thromb Haemost 2018 118 01 152 160 29304535 10.1160/TH17-06-0397

[71] Constantinescu-Bercu A Smith K E Wong S Y Von Willebrand factor deficiency impairs angiogenesis via angiopoietin-2: relevance for gut angiodysplasia Blood 2026 147 21 2541 2553 41587100 10.1182/blood.2025031558

[72] Zhang Y Qin D Shou A C Liu Y Wang Y Zhou L Exploring CAR-T cell therapy side effects: mechanisms and management strategies J Clin Med 2023 12 19 6124 37834768 10.3390/jcm12196124 PMC10573998

[73] Brudno J N Kochenderfer J N Current understanding and management of CAR T cell-associated toxicities Nat Rev Clin Oncol 2024 21 07 501 521 38769449 10.1038/s41571-024-00903-0 PMC11529341

[74] Li J Chen H Xu C Hu M Li J Chang W Systemic toxicity of CAR-T therapy and potential monitoring indicators for toxicity prevention Front Immunol 2024 15 1.422591E6 10.3389/fimmu.2024.1422591 PMC11381299 39253080

[75] Lindo L Wilkinson L H Hay K A Befriending the hostile tumor microenvironment in CAR T-cell therapy Front Immunol 2021 11 618387 33643299 10.3389/fimmu.2020.618387 PMC7902760

[76] Lee D W Gardner R Porter D L Current concepts in the diagnosis and management of cytokine release syndrome Blood 2014 124 02 188 195 24876563 10.1182/blood-2014-05-552729 PMC4093680

[77] Neelapu S S Tummala S Kebriaei P Chimeric antigen receptor T-cell therapy - assessment and management of toxicities Nat Rev Clin Oncol 2018 15 01 47 62 28925994 10.1038/nrclinonc.2017.148 PMC6733403

[78] Xiao X Huang S Chen S Mechanisms of cytokine release syndrome and neurotoxicity of CAR T-cell therapy and associated prevention and management strategies J Exp Clin Cancer Res 2021 40 01 367 34794490 10.1186/s13046-021-02148-6 PMC8600921

[79] Cao W J Niiya M Zheng X W Shang D Z Zheng X L Inflammatory cytokines inhibit ADAMTS13 synthesis in hepatic stellate cells and endothelial cells J Thromb Haemost 2008 6 07 1233 1235 18433458 10.1111/j.1538-7836.2008.02989.x PMC2582585

[80] Zegeye M M Lindkvist M Fälker K Activation of the JAK/STAT3 and PI3K/AKT pathways are crucial for IL-6 trans-signaling-mediated pro-inflammatory response in human vascular endothelial cells Cell Commun Signal 2018 16 01 55 30185178 10.1186/s12964-018-0268-4 PMC6125866

[81] Demosthenous C Evangelidis P Gatsis A Endothelial injury following CAR-T cell immunotherapy for hematological malignancies Cancers 2025 17 17 2876 40940973 10.3390/cancers17172876 PMC12427513

[82] Brudno J N Kochenderfer J N Recent advances in CAR T-cell toxicity: mechanisms, manifestations and management Blood Rev 2019 34 45 55 30528964 10.1016/j.blre.2018.11.002 PMC6628697

[83] Gu T Hu K Si X Hu Y Huang H Mechanisms of immune effector cell-associated neurotoxicity syndrome after CAR-T treatment WIREs Mech Dis 2022 14 06 e1576 35871757 10.1002/wsbm.1576 PMC9787013

[84] Fatahichegeni M Ansarian M A Wang Y Ren J Zhang T Wang X Immune effector cell-associated neurotoxicity syndrome following CAR T-cell therapy: a review of recent advances J Transl Med 2025 24 01 114 41454396 10.1186/s12967-025-07646-1 PMC12849438

[85] Genoud V Migliorini D Novel pathophysiological insights into CAR-T cell associated neurotoxicity Front Neurol 2023 14 1.108297E6 10.3389/fneur.2023.1108297 PMC10031128 36970518

[86] Pinto S N Krenciute G The mechanisms of altered blood-brain barrier permeability in CD19 CAR T-cell recipients Int J Mol Sci 2024 25 01 644 38203814 10.3390/ijms25010644 PMC10779697

[87] Shoaib A Gralnek S Lutfi F Abdelhakim H Zheng X L Pre-infusion ADAMTS13 and VWF levels are associated with clinical outcomes of patients receiving CAR-T cell therapy Blood 2025 146(suppl 1)2339

[88] Teachey D T Lacey S F Shaw P A Identification of predictive biomarkers for cytokine release syndrome after chimeric antigen receptor T-cell therapy for acute lymphoblastic leukemia Cancer Discov 2016 6 06 664 679 27076371 10.1158/2159-8290.CD-16-0040 PMC5448406

[89] Norelli M Camisa B Barbiera G Monocyte-derived IL-1 and IL-6 are differentially required for cytokine-release syndrome and neurotoxicity due to CAR T cells Nat Med 2018 24 06 739 748 29808007 10.1038/s41591-018-0036-4

[90] Jain M D Smith M Shah N N How I treat refractory CRS and ICANS after CAR T-cell therapy Blood 2023 141 20 2430 2442 36989488 10.1182/blood.2022017414 PMC10329191

[91] Wei Z Xu J Zhao C Prediction of severe CRS and determination of biomarkers in B cell-acute lymphoblastic leukemia treated with CAR-T cells Front Immunol 2023 14 1.273507E6 10.3389/fimmu.2023.1273507 PMC10579557 37854590

[92] Nawas M T Sanchez-Escamilla M Devlin S M Dynamic EASIX scores closely predict nonrelapse mortality after allogeneic hematopoietic cell transplantation Blood Adv 2022 6 22 5898 5907 35977079 10.1182/bloodadvances.2022007381 PMC9661383

[93] Penack O Luft T Peczynski C Endothelial activation and stress index (EASIX) to predict mortality after allogeneic stem cell transplantation: a prospective study J Immunother Cancer 2024 12 01 e007635 38199608 10.1136/jitc-2023-007635 PMC10806535

[94] Muratore E Gambuti G Leardini D The EASIX score as a predictor of sinusoidal obstruction syndrome and nonrelapse mortality in paediatric patients receiving allogeneic haematopoietic stem cell transplantation Bone Marrow Transplant 2025 60 03 346 352 39658654 10.1038/s41409-024-02489-8 PMC11893459

[95] Heying R Nürnberger W Spiekerkötter U Göbel U Hepatic veno-occlusive disease with severe capillary leakage after peripheral stem cell transplantation: treatment with recombinant plasminogen activator and C1-esterase inhibitor concentrate Bone Marrow Transplant 1998 21 09 947 949 9613790 10.1038/sj.bmt.1701211

[96] Carreras E Diaz-Ricart M The role of the endothelium in the short-term complications of hematopoietic SCT Bone Marrow Transplant 2011 46 12 1495 1502 21460864 10.1038/bmt.2011.65

[97] Nakamae H Yamane T Hasegawa T Risk factor analysis for thrombotic microangiopathy after reduced-intensity or myeloablative allogeneic hematopoietic stem cell transplantation Am J Hematol 2006 81 07 525 531 16755559 10.1002/ajh.20648

[98] Ortí G Dachy G Graham C E Less frequent complications following CAR T-cell therapies: hemophagocytic lymphohistiocytosis, graft-versus-host disease, thrombotic microangiopathy, coagulation disorders and secondary malignancies: best practice recommendations from the EBMT practice harmonization and guidelines committee Bone Marrow Transplant 2025 60 06 751 758 40205032 10.1038/s41409-025-02567-5

[99] Tomasik J Avni B Grisariu S Endothelial activation and stress index score as a prognostic factor of cytokine release syndrome in CAR-T patients - a retrospective analysis of multiple myeloma and large B-cell lymphoma cohortsArch Immunol Ther Exp2024 72(01) 10.2478/aite-2024-0018 39277881

[100] Galli E Sorà F Hohaus S Endothelial activation predicts disseminated intravascular coagulopathy, cytokine release syndrome and prognosis in patients treated with anti-CD19 CAR-T cells Br J Haematol 2023 201 01 86 94 36503182 10.1111/bjh.18596

[101] Zhao Y Zhang X Zhang M Modified EASIX scores predict severe CRS/ICANS in patients with acute myeloid leukemia following CLL1 CAR-T cell therapy Ann Hematol 2024 103 03 969 980 38214708 10.1007/s00277-024-05617-y

[102] Zandaki D Selukar S Bi Y EASIX and m-EASIX predict CRS and ICANS in pediatric and AYA patients after CD19-CAR T-cell therapy Blood Adv 2025 9 02 270 279 39325974 10.1182/bloodadvances.2024014027 PMC11782822

[103] Gust J Finney O C Li D Glial injury in neurotoxicity after pediatric CD19-directed chimeric antigen receptor T cell therapy Ann Neurol 2019 86 01 42 54 31074527 10.1002/ana.25502 PMC9375054

[104] Weinkove R George P Dasyam N McLellan A D Selecting costimulatory domains for chimeric antigen receptors: functional and clinical considerations Clin Transl Immunol 2019 8 05 e1049 10.1002/cti2.1049 PMC6511336 31110702

[105] Cook M S King E Flaherty K R CAR-T cells containing CD28 versus 4-1BB co-stimulatory domains show distinct metabolic profiles in patients Cell Rep 2025 44 07 115973 40650909 10.1016/j.celrep.2025.115973

[106] Cappell K M Kochenderfer J N A comparison of chimeric antigen receptors containing CD28 versus 4-1BB costimulatory domains Nat Rev Clin Oncol 2021 18 11 715 727 34230645 10.1038/s41571-021-00530-z

[107] Wang Y Qi K Cheng H Coagulation disorders after chimeric antigen receptor T cell therapy: analysis of 100 patients with relapsed and refractory hematologic malignancies Biol Blood Marrow Transplant 2020 26 05 865 875 31786240 10.1016/j.bbmt.2019.11.027

[108] Shao M Yu Q Teng X CRS-related coagulopathy in BCMA targeted CAR-T therapy: a retrospective analysis in a phase I/II clinical trial Bone Marrow Transplant 2021 56 07 1642 1650 33608658 10.1038/s41409-021-01226-9

[109] Jiang H Liu L Guo T Improving the safety of CAR-T cell therapy by controlling CRS-related coagulopathy Ann Hematol 2019 98 07 1721 1732 31055613 10.1007/s00277-019-03685-z

[110] Dong R Wang Y Lin Y The correlation factors and prognostic significance of coagulation disorders after chimeric antigen receptor T cell therapy in hematological malignancies: a cohort study Ann Transl Med 2022 10 18 975 36267762 10.21037/atm-22-3814 PMC9577724

[111] Peng X Zhang X Zhao M Coagulation abnormalities associated with CAR-T-cell therapy in haematological malignancies: a review Br J Haematol 2024 205 02 420 428 38887101 10.1111/bjh.19583

[112] Buechner J Grupp S A Hiramatsu H Practical guidelines for monitoring and management of coagulopathy following tisagenlecleucel CAR T-cell therapy Blood Adv 2021 5 02 593 601 33496754 10.1182/bloodadvances.2020002757 PMC7839371

[113] Johnsrud A Craig J Baird J Incidence and risk factors associated with bleeding and thrombosis following chimeric antigen receptor T-cell therapy Blood Adv 2021 5 21 4465 4475 34521106 10.1182/bloodadvances.2021004716 PMC8579267

[114] Jess J Yates B Dulau-Florea A CD22 CAR T-cell associated hematologic toxicities, endothelial activation and relationship to neurotoxicity J Immunother Cancer 2023 11 06 e005898 37295816 10.1136/jitc-2022-005898 PMC10277551

[115] Perna F Parekh S Diorio C CAR T-cell toxicities: from bedside to bench, how novel toxicities inform laboratory investigations Blood Adv 2024 8 16 4348 4358 38861351 10.1182/bloodadvances.2024013044 PMC11375260

[116] Bindal P Patell R Chiasakul T A meta-analysis to assess the risk of bleeding and thrombosis following chimeric antigen receptor T-cell therapy: communication from the ISTH SSC subcommittee on Hemostasis and malignancy J Thromb Haemost 2024 22 07 2071 2080 38574863 10.1016/j.jtha.2024.03.021 PMC11437522

[117] Hashmi H Mirza A S Darwin A Venous thromboembolism associated with CD19-directed CAR T-cell therapy in large B-cell lymphoma Blood Adv 2020 4 17 4086 4090 32877523 10.1182/bloodadvances.2020002060 PMC7479945

[118] Parks A L Kambhampati S Fakhri B Incidence, management and outcomes of arterial and venous thrombosis after chimeric antigen receptor modified T cells for B cell lymphoma and multiple myeloma Leuk Lymphoma 2021 62 04 1003 1006 33258699 10.1080/10428194.2020.1852474 PMC8012226

[119] Schorr C Forindez J Espinoza-Gutarra M Mehta R Grover N Perna F Thrombotic events are unusual toxicities of chimeric antigen receptor T-cell therapies Int J Mol Sci 2023 24 09 8349 37176053 10.3390/ijms24098349 PMC10179014

[120] Yamasaki-Morita M Arai Y Ishihara T Relative hypercoagulation induced by suppressed fibrinolysis after tisagenlecleucel infusion in malignant lymphoma Blood Adv 2022 6 14 4216 4223 35580321 10.1182/bloodadvances.2022007454 PMC9327547

[121] Abufarhaneh M Pandya R Lazo-Langner A Iansavitchene A Dematagoda D Risk of bleeding and thrombosis with CAR T-cell therapy: a scoping review Blood 2023 142(suppl 1)5547

[122] Qi J Lv X Chen J TNF-α increases the risk of bleeding in patients after CAR T-cell therapy: a bleeding model based on a real-world study of Chinese CAR T working party Hematol Oncol 2022 40 01 63 71 34606093 10.1002/hon.2931

[123] Lee D Danielian P Leader A Bleeding and thrombosis in patients receiving chimeric antigen receptor T-cell therapy Bleeding Thromb Vasc Biol 2026 10.4081/btvb.2026.461

[124] Bailey A Zhou S Q Tagalakis V Incidence and risk factors for venous thromboembolism after CAR T-cell therapy: a systematic review and meta-analysis J Clin Oncol 2025 43 16 e24085

[125] Melo Garcia L Wang T-F Kennah M Incidence of thrombosis and bleeding in patients receiving CAR-T cell therapy: a multicentric retrospective analysis of the Canadian experience Blood 2025 146(suppl 1)4496

[126] Galli E Sorà F Rossi E Coagulopathy in CAR-T: critical concern or mere blip? Br J Haematol 2024 205 02 409 410 38938123 10.1111/bjh.19619

[127] Schoettler M L Carreras E Cho B Harmonizing definitions for diagnostic criteria and prognostic assessment of transplantation-associated thrombotic microangiopathy: a report on behalf of the European Society for Blood and Marrow Transplantation, American Society for Transplantation and Cellular Therapy, Asia-Pacific blood and marrow transplantation group, and Center for International Blood and Marrow Transplant Research Transplant Cell Ther 2023 29 03 151 163 36442770 10.1016/j.jtct.2022.11.015 PMC10119629

[128] Giavridis T van der Stegen S JC Eyquem J Hamieh M Piersigilli A Sadelain M CAR T cell-induced cytokine release syndrome is mediated by macrophages and abated by IL-1 blockade Nat Med 2018 24 06 731 738 29808005 10.1038/s41591-018-0041-7 PMC6410714

[129] Yu S L Abdulhaleem M Ul Abedin M S The effect of statin use on outcome in patients receiving chimeric antigen receptor T-cell therapy. Transplantation and cellular therapy Off Publ Am Soc Transplant Cell Therap 2025 31 02 S247

[130] Jacobson C A Rosenthal A C Arnason J A phase 2 trial of defibrotide for the prevention of chimeric antigen receptor T-cell-associated neurotoxicity syndrome Blood Adv 2023 7 21 6790 6799 37399456 10.1182/bloodadvances.2023009961 PMC10692301

[131] Chen Y Li R Shang S Therapeutic potential of TNFα and IL1β blockade for CRS/ICANS in CAR-T therapy via ameliorating endothelial activation Front Immunol 2021 12 623610 34093519 10.3389/fimmu.2021.623610 PMC8170323

[132] Stephen L Y Muhammad S U Nolan H The effect of statin use on outcome in patients receiving chimeric antigen receptor T-cell therapy Transplant Cell Ther. 2025 31 02 S247

[133] Bendapudi P K Foy B H Mueller S B Recombinant ADAMTS13 for immune thrombotic thrombocytopenic purpura N Engl J Med 2024 390 18 1690 1698 38718359 10.1056/NEJMoa2402567 PMC11128310

[134] Scully M Cataland S R Peyvandi F Caplacizumab treatment for acquired thrombotic thrombocytopenic purpura N Engl J Med 2019 380 04 335 346 30625070 10.1056/NEJMoa1806311

[135] Turecek P L Peck R C Rangarajan S Recombinant ADAMTS13 reduces abnormally up-regulated von Willebrand factor in plasma from patients with severe COVID-19 Thromb Res 2021 201 100 112 33662796 10.1016/j.thromres.2021.02.012 PMC7890348

[136] Darwish I Liles W C Emerging therapeutic strategies to prevent infection-related microvascular endothelial activation and dysfunction Virulence 2013 4 06 572 582 23863603 10.4161/viru.25740 PMC5359747

[137] Kim D H Jung Y J Lee A S COMP-angiopoietin-1 decreases lipopolysaccharide-induced acute kidney injury Kidney Int 2009 76 11 1180 1191 19812542 10.1038/ki.2009.387

[138] McCarter S D Mei S H Lai P F Cell-based angiopoietin-1 gene therapy for acute lung injury Am J Respir Crit Care Med 2007 175 10 1014 1026 17322110 10.1164/rccm.200609-1370OC

[139] Witzenbichler B Westermann D Knueppel S Schultheiss H P Tschope C Protective role of angiopoietin-1 in endotoxic shock Circulation 2005 111 01 97 105 15611372 10.1161/01.CIR.0000151287.08202.8E

[140] Kumpers P Gueler F David S The synthetic Tie2 agonist peptide vasculotide protects against vascular leakage and reduces mortality in murine abdominal sepsis Crit Care 2011 15 05 R261 22040774 10.1186/cc10523 PMC3334812

[141] David S Ghosh C C Kümpers P Effects of a synthetic PEG-ylated Tie-2 agonist peptide on endotoxemic lung injury and mortality Am J Physiol Lung Cell Mol Physiol 2011 300 06 L851 L862 21421750 10.1152/ajplung.00459.2010 PMC3119126

[142] Alfieri A Watson J J Kammerer R A Angiopoietin-1 variant reduces LPS-induced microvascular dysfunction in a murine model of sepsis Crit Care 2012 16 05 R182 23036162 10.1186/cc11666 PMC3682284

